# A Worm’s Eye View

**DOI:** 10.3201/eid2408.AC2408

**Published:** 2018-08

**Authors:** Byron Breedlove, Richard Bradbury

**Affiliations:** Centers for Disease Control and Prevention, Atlanta, Georgia, USA

**Keywords:** art science connection, emerging infectious diseases, art and medicine, about the cover, Ben Taylor, The Host, A Worm’s Eye View, bacteria, neglected tropical diseases, parasitic diseases, parasites, Loa loa, loiasis, African eyeworm, hookworms, strongyloides, public health

**Figure:**
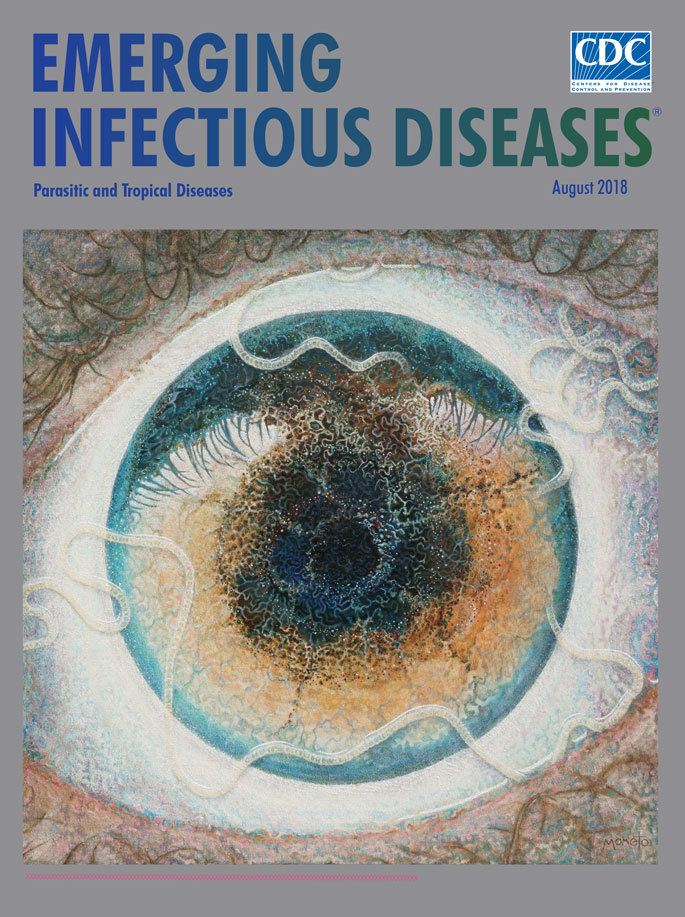
**Ben Taylor (aka Mometo) (1970–), The Host (2014).** Oil and textile on canvas.39.4 in × 39.4 in/100 cm × 100 cm). Image used by permission of the artist. Dartmoor, United Kingdom.

Seeing a several-centimeters-long worm traversing the conjunctiva of an eye is often the moment when many people realize they are infected with *Loa loa*, commonly called the African eyeworm, a parasitic nematode that migrates throughout the subcutaneous and connective tissues of infected persons. Infection with this worm is called loiasis and is typically diagnosed either by the worm’s appearance in the eye or by a history of localized Calabar swellings, named for the coastal Nigerian town where that symptom was initially observed among infected persons. Endemic to a large region of the western and central African rainforests, the *Loa loa* microfilariae are passed to humans primarily from bites by flies from two species of the genus *Chrysops*, *C. silacea* and *C. dimidiate*. The more than 29 million people who live in affected areas of Central and West Africa are potentially at risk of loiasis. 

Researcher Chris Desjardins notes that “while *Loa loa* does less damage than a few of its filarial cousins, sometimes causing pain or swelling under the skin, it is still ‘horrifying’ since the worm often goes unnoticed until it enters the eye.” Count contemporary artist Ben Taylor among those who discovered he was a host for *Loa loa* when he spotted one of the worms in his eye. 

Taylor, who was born in Australia and grew up in Africa and Scotland, was an industrial model maker before becoming a computer-generated imagery artist. He eventually left the corporate world to create sculptural works and paintings, the former influenced by his time spent as a model maker, the latter by his work as a computer-generated imagery artist (B. Taylor, pers. comm., 2018 June 29). Taylor also has, in his words, “spent a lifetime living and travelling in far flung lands.”

In 2013, Taylor visited the jungles of Gabon for several weeks. After his return to England, he started, but could not complete, an elaborate abstract painting that involved meticulously scraping the wet paint to reveal the underlying dry layers, “forming long undulating lines that, with hindsight, had a distinctly wormy look and feel” (B. Taylor, pers. comm., 2018 June 29). As Taylor notes on his website, “I was not able to fully bond with the piece at that time, it was a struggle to complete and I was not satisfied with the end result. I had no idea what compelled me to paint it, or what it was trying to say, and it rested in this unsatisfactory state for many months.”I was not able to fully bond with the piece at that time, it was a struggle to complete and I was not satisfied with the end result. I had no idea what compelled me to paint it, or what it was trying to say, and it rested in this unsatisfactory state for many months.

At that time, Taylor was plagued by health problems that were proving challenging to diagnose or treat, including what he described as a blinding transient pain in his eye, which would recur and disappear. About 4 months after Taylor had set aside this painting, he witnessed something wriggling in his eye and promptly sought medical care, which resulted in an eye surgeon extracting a *Loa loa* worm from Taylor’s eye. Taylor notes that this event “was the start of new adventures as a medical novelty exhibit” during his week-long stay at the London Hospital for Tropical Diseases for “an intensive bout of treatment.” 

Taylor discovered that he had returned from this travels not just with *Loa loa* but with two additional parasitic diseases, hookworm infection and strongyloidiasis. Finally having a diagnosis for his ongoing health issues was tinged with an awareness of “how serious the treatment was likely to be” (pers. comm., 2018 June 29). 

While he was recovering from this trio of parasitic infections and related treatments, Taylor rediscovered the unfinished painting stashed upside down in his studio: “I immediately saw that what I had painted was an eye made out of worms. Out came the oil paints, and a few days later, the painting was finished.” The artist dubbed his finished work “The Host,” perhaps his way of winking at the adversity he experienced in providing a home for this parasite.

The translucent white ribbon that coils across the conjunctiva and pupil dominates the image on the canvas and seems to rise above its surface. Though it is natural to gaze at the dark blue center of the eye, the viewer’s attention keeps returning to the disturbing pale segmented shapes. A constellation of wormlike patterns that swirl toward the center of the eye is rendered with detailed density. The completed work depicts what the artist describes as his “descent into sickness and desperation as no diagnosis was forthcoming for my illness.” 

Loiasis is believed to affect an estimated 12 million people in regions where it is endemic. In many of those places, the public health and medical infrastructure and expertise essential for its successful diagnosis and treatment are lacking. The compelling artwork on this month’s cover may help boost awareness concerning the public health burden posed by this overlooked tropical parasitic infection. 
